# Clinical Results after Release of Sternocleidomastoid Muscle Surgery for Neglected Congenital Muscular Torticollis—Unipolar vs. Bipolar Release Surgery

**DOI:** 10.3390/jcm13010131

**Published:** 2023-12-26

**Authors:** Haruki Funao, Norihiro Isogai, Nao Otomo, Kento Yamanouchi, Ryo Mizukoshi, Naruhito Fujita, Shigeto Ebata, Ken Ishii, Mitsuru Yagi

**Affiliations:** 1 Department of Orthopaedic Surgery, School of Medicine, International University of Health and Welfare, Narita 286-8686, Japan; n.isogai0813@gmail.com (N.I.); naoootomo@yahoo.co.jp (N.O.); yamaken0331@gmail.com (K.Y.); ryomizukoshi.0817@gmail.com (R.M.); naruhito88@hotmail.com (N.F.); ebatas310@gmail.com (S.E.); 2 Department of Orthopaedic Surgery, International University of Health and Welfare Narita Hospital, Narita 286-0124, Japan; 3 Department of Orthopaedic Surgery, International University of Health and Welfare Mita Hospital, Tokyo 108-8329, Japan; 4 Department of Orthopaedic Surgery, Keio University School of Medicine, Tokyo 160-0016, Japan; kenishii88@gmail.com; 5 Society for Minimally Invasive Spinal Treatment (MIST), Tokyo 101-0063, Japan

**Keywords:** congenital muscular torticollis, neglected congenital muscular torticollis, bipolar release of sternocleidomastoid muscle, unipolar release of sternocleidomastoid muscle, surgical outcome

## Abstract

Background: Although the surgical release of the sternocleidomastoid muscle (SCM) is required for residual congenital muscular torticollis (CMT), the surgical outcomes between bipolar and unipolar SCM release remained unclear. The purpose of the present study was to assess surgical outcomes after bipolar and unipolar release of SCM in adolescent/adult patients with neglected CMT. Methods: Twenty-one consecutive adolescent/adult patients with neglected CMT who underwent surgical treatment were enrolled. Clinical and radiographic outcomes were evaluated at preoperative and final follow-up. Results: The bipolar release of SCM was performed in 3 patients (B group; males, *n* = 1, females, *n* = 2) and the unipolar release of SCM in 18 patients (U group; males, *n* = 6, females, *n* = 12). The mean age at surgery was 40.0 ± 17.0 years in Group B, and that was 32.3 ± 13.1 years in Group U (*p* = 0.47). The mean follow-up period was 16.0 ± 5.7 months in Group B and 10.4 ± 7.6 months in Group U (*p* = 0.22). Cervicomandibular angle correction rates were comparable at 75.4 ± 2.4% for the B group and 73.1 ± 11.7% for the U group (*p* = 0.62). There was no significant difference in clinical outcome according to the modified Cheng and Tang score between the two groups (*p* = 0.89). No major complications arose, although one patient exhibited a transient neurological deficit of the greater auricular nerve, and one patient developed a hematoma in the B group. Conclusions: The unipolar SCM release appeared to be a non-inferiority and less invasive procedure, minimizing surgical scars and avoiding potential auricular nerve damage in adolescent/adult patients with neglected CMT.

## 1. Introduction

Congenital muscular torticollis (CMT) occurs at a frequency of 0.3% to 2% [1], with a higher incidence in cases of breech deliveries and dystocia [2,3]. CMT is generally noticed and diagnosed in infancy through parental observation or during infant physical examination when the head is found to be tilted. Tumor-like palpation or tightening and shortening of an affected sternocleidomastoid muscle (SCM) are observed. CMT is more common on the right side, with a male-to-female ratio of 3:2 [4]. 

The diagnosis of neglected CMT is usually made by interview, visual examination, palpation, evaluation of cervical spine range of motion, and imaging studies such as simple X-rays and ultrasound echograms. Palpation should be performed bilaterally, and in infants, an elastic hard mass is palpable on the SCM on the affected side. In infancy and later, the SCM would be palpated for thickening, hardness, or shortening. In some cases, in which the clavicular branch was mainly tense, it was difficult to diagnose because the oblique neck was less noticeable at first glance. The tightening and thickening of the SCM were noticeable when the patient’s neck bent or rotated to the healthy side, which is useful for diagnosis. In general, rotation is restricted to the affected side, and side-bending is restricted to the healthy side. Radiographic findings show that the cervical spine is generally tilted toward the healthy side, and the head is tilted toward the affected side. A computed tomography axial image shows findings similar to type 1 of the Fielding classification [5], so care must be taken to differentiate this from atlantoaxial rotatory fixation [6]. If the patient has neurological findings, or if it is deemed necessary for some other reason, magnetic resonance imaging should be obtained to confirm degenerative changes, narrowing of the spinal canal or foramen, tumors, or infection.

More than 80% of the cases heal spontaneously or with conservative treatment, such as stretching, by the age of 1 year [4,7]. Treatment for CMT includes lifestyle guidance, stretching and other rehabilitation, orthosis, or surgery. Rehabilitation therapy such as stretching has been reported to be useful in infancy [8], and recently, it has been reported that adding microwaves to conventional conservative treatment has improved torticollis and range of motion and shortened treatment time [9]. However, when conservative treatment fails in residual CMT, those cases should be treated by surgery before school age because surgical treatment after the age of 5 years may result in head and face deformities and sternocleidomastoid scarring [10]. It has been reported that irreversible head and facial deformities remain after the age of 10 years [11]. Unfortunately, some residual CMTs are left behind and untreated, resulting in neglected CMT. Although craniofacial deformity would remain in neglected CMT, it is reported that surgical treatment is effective in improving appearance and function even after 15 years old [12]. 

To date, various surgical procedures have been reported in child or adult patients, including bipolar or unipolar release of SCM [13,14], Z lengthening [15], and partial resection of SCM [16]. Z-lengthening is not recommended for child patients due to their subsequent growth, resulting in the tethering of SCM and the recurrence of CMT [15]. Although unipolar release of SCM was reported to be successful in most cases, reoperation or additional bipolar release of SCM may be required due to recurrence [2,13]. Previous studies recommended bipolar release of SCM for patients over 6 years old with neglected CMT or recurrent cases [2,17]. However, surgical outcomes between bipolar and unipolar releases of SCMs still remain unclear. To the best of our knowledge, there were no comparative studies of bipolar and unipolar release of SCM in adolescent/adult patients with neglected CMT. Here, we assessed clinical and radiographic outcomes after bipolar and unipolar releases of SCMs in adolescent/adult patients with neglected CMT. We hypothesized that unipolar release of SCM was sufficient for neglected CMT, even in adolescent/adult patients and recurrent cases.

## 2. Materials and Methods

This is a retrospective case-series study. The inclusion criteria were as follows: the diagnosis of neglected CMT was confirmed by interview, visual examination, palpation, evaluation of cervical spine range of motion, and imaging studies; symptomatic and residual CMT that required surgical treatment; and age at surgery was over 13 years old. Patients whose follow-up periods were less than 2 months after surgery were excluded from this study. All the patients had already been seen by previous doctors. Most of the patients were referred or visited to our hospital by information on the internet, and surgical treatments were recommended after lengthy discussions. The patients were advised both of bipolar and unipolar release of SCM. The patients were explained that bipolar release of SCM was a recommended procedure for neglected CMT based on the previous literature, and unipolar release of SCM was a possible procedure for obtaining enough cervical mobility based on our experience. Procedures were decided and performed according to the patient’s preferences. After surgery, all patients wore cervical collars at rest for 1–2 weeks, and they received physical therapy with a cervical range of motion training from the early postoperative period. The patients were advised to continue the range of motion training at least 3 months after surgery.

Age, gender, affected side of CMT, history of previous surgery for CMT, follow-up period, operative time, blood loss, radiographic parameters, modified Cheng and Tang scoring system [13], and complications were assessed and compared between bipolar release and unipolar release of SCM.

### 2.1. Surgical Procedures

Surgery was performed under general anesthesia in the supine position. The cervical spine was flexed backward with a shoulder pillow and rotated to the healthy side. An ultrasound echogram was useful for determining the location of skin incisions and identifying vessels and adjacent organs. Compared to the homogenous muscles and tendons of SCM on the healthy side, those on the affected side often appeared as a rounded, scar-like shadow on an ultrasound echogram. Identification of the clavicular branches was relatively easy in cases of CMT, in which the clavicular branch was mainly tense. In contrast, in some cases with a prominent sternal protrusion, the clavicular branch was not obvious; therefore, the incision site might be difficult to determine. In those cases, a preoperative echogram could identify the medial and lateral aspects of the clavicle branch; thus, it might be helpful in determining the incision site for the clavicle branch. The skin incisions were about 1.5 to 2 cm for each of the sternal and clavicular branches (two incisions) or 4 to 5 cm between the sternal and clavicular branches (one incision).

The release of the lower end of the sternal branch or clavicular branch was performed according to the more tense branch of the SCM. Once one of the lower ends of the SCM branches was identified, gauze was placed in the deep layer in order to lift the SCM branch, and the resection margin was marked with colored thread for resection of approximately 2 cm of the lower end of the SCM branch. This allowed the muscle tendon to be resected at the planned length. Without this marking, the planned resection length might not be gained because the muscle–tendon is shortened during the cut. While making sure that the muscle–tendon did not contain any vessels or nerve elements, the muscle–tendon was cut in a shallow and sequential fashion with either an electrocautery scalpel or bipolar hemostatic forceps. Once one of the lower ends of SCM branches had been completely dissected, the other branch was observed while lateral bending, rotating, or extension of the cervical spine. It is important to check the mobility of the cervical spine before and after one of the lower ends of SCM branches is dissected. Once the newly protruding other branches of SCM were identified, they were also dissected. The author proceeded with the dissection while frequently checking the mobility of the cervical spine, and the endpoint of the procedure was when sufficient mobility was obtained. In recurrent cases, the surrounding tissues have also become scarred; thus, dissection of platysma muscle or pretrachealis fasciae cervicalis may be required in some cases. The upper end of SCM could be released after dissection of the lower ends of SCM branches. Although we performed bipolar release of SCM in three cases based on the patients’ preferences in this study, we speculated that unipolar release of SCM could obtain enough mobility of the cervical spine even in adolescent/adult neglected CMT patients.

The surgical field was thoroughly washed out, and the bleeding should be adequately stopped with bipolar hemostatic forceps or hemostatic agents. Even if bleeding appears to have been sufficiently stopped, it may bleed again postoperatively, resulting in a subcutaneous hematoma. The author has experienced a large hematoma that took several weeks to absorb. If necessary, a drain can be placed. The gelatin sponge is sometimes placed to prevent reattachment of SCM; however, it can be a source of infection and is not considered useful in preventing reattachment of SCM, according to our experiences.

This study complied with the Declaration of Helsinki and was approved by the Ethics Review Committees of the International University of Health and Welfare Mita (approval number: 5-9-21) and Narita Hospital (approval number: 20-Nr-013).

### 2.2. Outcome Measurements

**Figure 1:**
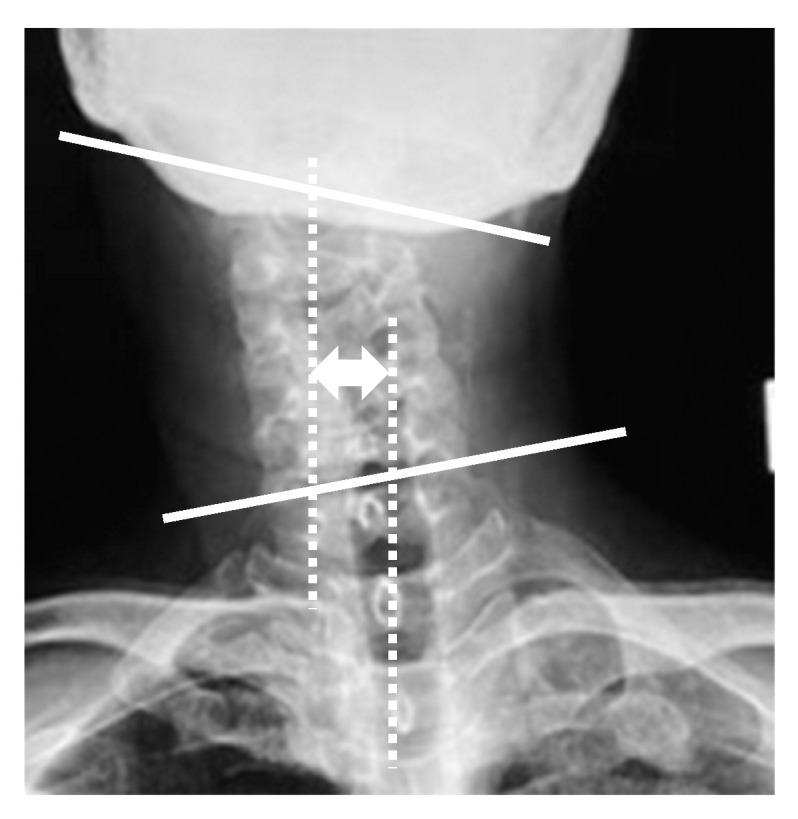
Radiographic parameters: cervicomandibular angle and C2 to T1 distance.

**Figure 2:**
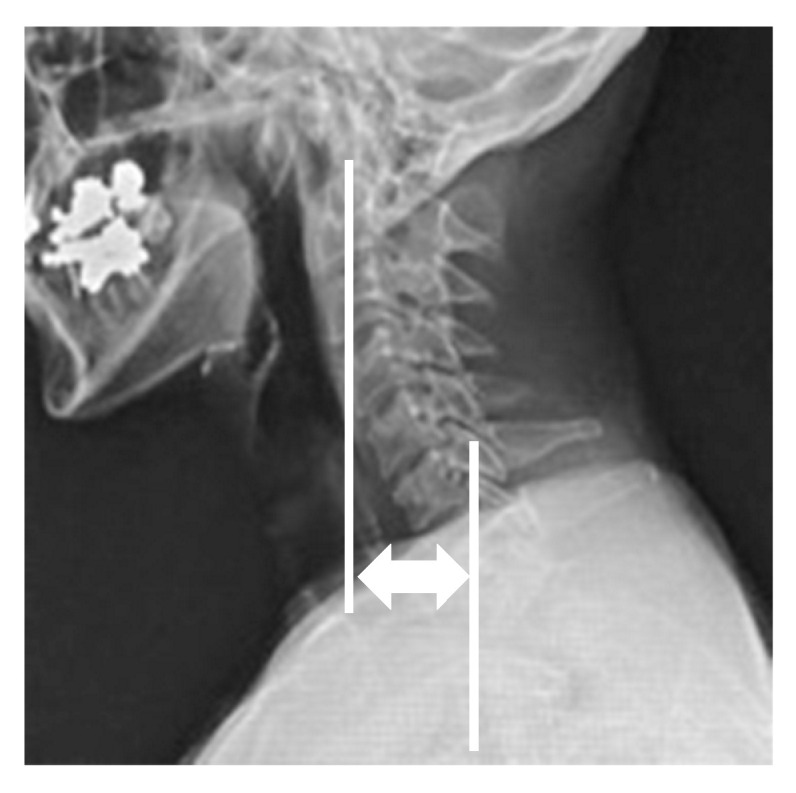
Radiographic parameters; C2–7 sagittal vertical axis.

Radiographic parameters included: cervicomandibular angle (CMA) for head tilt; C2 to T1 distance for lateral shift of neck; clavicle angle for shoulder balance; central sacral vertical line (CSVL) for coronal global balance; C2–7 angles (neutral, flexion, extension) for cervical alignment and range of motion on the sagittal plane; C2–7 sagittal vertical axis (C2–7 SVA); and C7 SVA for whole spinopelvic sagittal alignment. CMA was defined as the angle between a line connecting the lower margins of the mandibular angle and the superior endplate of C7 on the anterior and posterior radiographs. The C2 to T1 distance was defined as the distance between the vertical line from the center of the C2 vertebra and that of the T1 vertebra on the anterior and posterior radiographs (Figure 1). C2–7 angles were measured by determining the C2–7 angles between the inferior endplate of C2 and the superior endplate of C7 vertebra in neutral, flexion, and extension positions on the lateral radiographs. The cervical range of motion was calculated by subtracting flexion from extension C2–7 angles. CSVL was defined as the distance between the vertical line from the midline of the sacrum and that of the C7 vertebra, and clavicle angle was defined as the angle between the horizontal line and tangential line connecting the highest two points of each clavicle on the standing anterior and posterior radiographs. C7 SVA was defined as the distance between the C7 plumb line and the posterior superior corner of the S1 vertebra, and C2–7 SVA was defined as the distance between the C2 plumb line and the posterior superior corner of the C7 vertebra on the standing lateral radiograph (Figure 2). Measurements were taken by three spine surgeons with more than 10 years of experience at our institution.

CMA was defined as the angle between a line connecting the lower margins of the mandibular angle and the superior endplate of C7 on the anterior and posterior radiographs (white lines). The C2 to T1 distance was defined as the distance between the vertical line from the center of the C2 vertebra and that of the T1 vertebra on the anterior and posterior radiographs (white dotted lines).

The C2–7 SVA was defined as the distance between the C2 plumb line and the posterior superior corner of the C7 vertebra on the standing whole spinopelvic radiograph (white lines).

A modified Cheng and Tang scoring system is used for quantifying clinical outcomes. This system consists of rotational deficits, side flexion deficits, craniofacial symmetry, scar, band, head tilt, appearance, and function, with 17 to 21 points being excellent, 12 to 16 points good, 7 to 11 points fair, and 6 or less points poor [13].

### 2.3. Statistical Analysis

All data were expressed as the mean ± standard deviation. The Mann–Whitney U test and Fisher’s exact test were used for statistical analyses. SPSS, version 29.0 (IBM Corp., Armonk, NY, USA), was used, and a *p*-value < 0.05 was considered statistically significant.

## 3. Results

### 3.1. Demographic Characteristics

Demographic characteristics are shown in Table 1. Twenty-one consecutive adolescent/adult patients with neglected CMT were enrolled. All patients complained of cosmetic problems and limitations in the range of motion of the cervical spine. Neck and/or shoulder stiffness and/or pain were observed in 15 patients, and numbness around the neck, shoulder, or upper extremities was observed in 9 patients. The affected sides were on the right side in 3/3 of patients in Group B, and those were on the right side in 12/18 patients in Group U (*p* = 0.53). Three patients who underwent bipolar release of SCM were divided into Group B, and 18 patients who underwent unipolar release of SCM were divided into Group U. The mean age was 40.0 ± 17.0 (16, 51, 53) years in Group B, and 32.3 ± 13.1 (13–59) years in group U. There were no significant differences in age at surgery, sex, height, weight, and body mass index between the two groups. The mean follow-up period was 16.0 ± 5.7 (12–24) months in Group B and 10.4 ± 7.6 (3–36) months in Group U (*p* = 0.22).

### 3.2. Surgical Data and Adverse Events

The mean operative time was 60.3 ± 9.7 (52–74) minutes in Group B and 51.1 ± 15.5 (33–86) minutes in Group U (*p* = 0.22). The mean amount of estimated blood loss was 7.0 ± 5.7 (3–15) mL in Group B, and that was 2.4 ± 1.7 (1–5) mL in Group U (*p* = 0.13). Operative time tended to be shorter, and blood loss tended to be lesser in Group U, although they did not reach significance.

Perioperative complications included transient auricular nerve palsy in one patient in Group B, which resolved approximately 1 month after surgery. This transient palsy appeared to be caused by intraoperative retraction. The postoperative hematoma was observed in one patient in Group B. The patient did not complain of difficulty breathing or anemia, and the hematoma was resolved in 2 weeks after surgery.

### 3.3. Radiographic Outcomes

Preoperative and postoperative radiographic parameters in both groups are shown in Table 1. CMA improved from 13.7 ± 2.1 (11–16)° preoperatively to 3.3 ± 0.5 (3–4)° postoperatively in Group B (*p* = 0.10), and that in Group U significantly improved from 15.4 ± 6.5 (7–27)° to 4.1 ± 2.6 (1–10)° (*p* < 0.01). The ratios of the difference between preoperative and postoperative angles per preoperative angle of CMA were 75.4 ± 2.4 (72.7–78.6) % in Group B and 73.1 ± 11.7 (55.6–91.7) % in Group U and there was no significant difference between the two groups (*p* = 0.62). The C2 to T1 distance improved from 12.0 ± 4.5 (6–17) mm to 3.3 ± 1.2 (2–5) mm in Group B (*p* = 0.10), and that in Group U significantly improved from 14.2 ± 7.7 (5–32) mm preoperatively to 5.2 ± 4.8 (1–18) mm postoperatively (*p* < 0.01). Cervical lordosis in extension position increased from 27.7 ± 11.7 (12–40)° to 42.7 ± 5.2 (38–50)° in Group B (*p* = 0.20), and that in Group U significantly increased from 33.6 ± 11.7 (17–59)° to 44.1 ± 12.3 (22–59)° (*p* < 0.05). Cervical range of motion increased from 42.0 ± 7.1 (32–48)° to 66.3 ± 11.1 (51–77)° in Group B (*p* = 0.10), and that significantly increased from 62.2 ± 12.2 (35–90)° to 71.5 ± 13.1 (48–92)° in U group (*p* < 0.05). Clavicle angle improved from 2.0 ± 1.6 (0–4)° to 1.3 ± 0.5 (1–2)° in Group B **(***p* = 0.70), and that in U group significantly improved from 2.7 ± 1.3 (1–4)° to 1.2 ± 0.5 (0–2)° (*p* < 0.01). CSVL decreased from 7.0 ± 5.4 (0–13) mm to 5.7 ± 2.6 (2–8) mm in Group B (*p* = 0.70), and that in the U group significantly decreased from 13.5 ± 10.3 (0–34) mm to 5.3 ± 2.8 (0–10)° (*p* < 0.05). C2–7 SVA improved from 28.7 ± 11.5 (14–42) mm to 14.7 ± 6.3 (6–21) mm in the B group (*p* = 0.40), and that in the U group significantly improved from 31.6 ± 11.4 (14–42) mm to 18.4 ± 8.1 (6–40) mm (*p* < 0.01) (Table 2).

### 3.4. Clinical Outcomes

According to the modified Cheng and Tang scoring system, two patients were classified as excellent and one patient as good in Group B, and eight patients were classified as excellent and ten patients as good in Group U (*p* = 0.59). The mean modified Cheng and Tang score was 16.0 ± 1.6 scores in group B and 15.7 ± 2.7 scores in group U. There was no significant difference between the two groups (*p* = 0.89). All patients improved in appearance complaints and range of motion limitation. Cervical or shoulder stiffness, neck pain, or scapula pain improved in 14/15 patients, and numbness in the upper extremities improved in all patients after surgery.

### 3.5. Representative Case of Bipolar Release of Sternocleidomastoid Muscle

**Figure 3:**
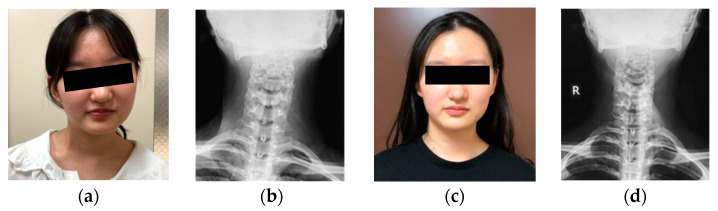

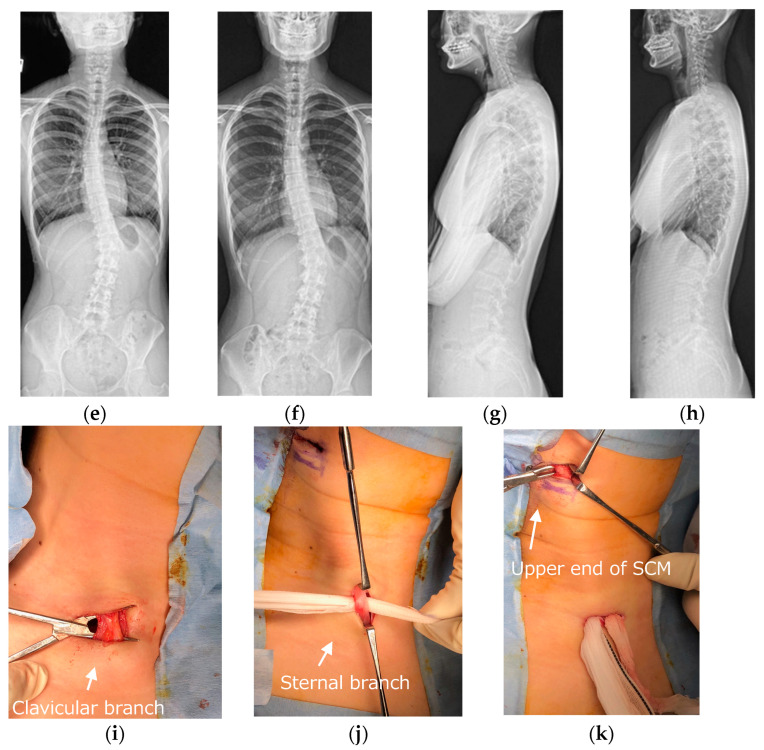
Sixteen-year-old female was treated with the bipolar release of the sternocleidomastoid. Pre- (**a**,**b**,**e**,**g**) and postoperative (**c**,**d**,**f**,**h**) appearance, radiographs, and intraoperative photographs. The head was markedly tilted to the right side, and the neck was shifted to the left side preoperatively. Right lower ends of the sternal branch and clavicular branch of the sternocleidomastoid (SCM) were released at one incision (**i**,**j**). The upper end of the SCM was released at the other incision (**k**). There were good improvements in appearance and postoperative X-rays after the bipolar release of SCM.

Sixteen-year-old female. She had a history of adolescent idiopathic scoliosis. She complained of head tilt and restriction of cervical range of motion, as well as neck pain and shoulder stiffness. She presented with a markedly thickened clavicular branch of the right SCM, especially in side bending to the left side. The patient underwent a bipolar release of SCM. Her radiographic parameters improved from baseline to final follow-up: CMA from 16° to 4°, C2 to T1 distance from 13 mm to 5 mm, clavicle angle from 4° to 1°, CSVL from 13 mm to 8 mm, cervical range of motion from 32° to 77°, and C2–7 SVA from 42 to 17 mm (Figure 3).

### 3.6. Representative Case of Unipolar Release of Sternocleidomastoid Muscle

**Figure 4:**
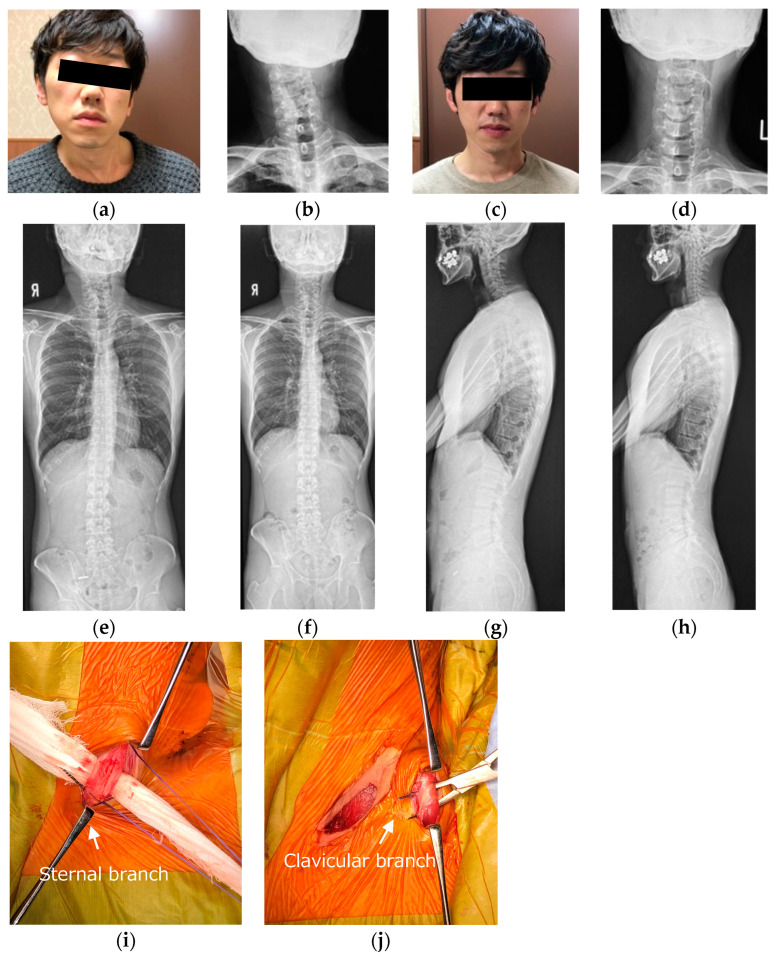
Forty-year-old male was treated with unipolar release of the sternocleidomastoid. Pre- (**a**,**b**,**e**,**g**) and postoperative (**c**,**d**,**f**,**h**) appearance, radiographs, and intraoperative photographs. The head was markedly tilted to the left side, and the neck was shifted to the right side preoperatively. Left lower ends of the sternal branch and clavicular branch of the SCM were released at two incisions (**i**,**j**). There were good improvements in appearance and postoperative X-rays after the unipolar release of SCM.

Forty-year-old male. He had no past medical history except for his neglected CMT. He complained of head tilt and restriction of cervical range of motion, as well as neck pain and shoulder stiffness. He presented with a marked head tilt and thickened sternal and clavicular branches of the left SCM. The patient underwent a unipolar release of SCM. His radiographic parameters improved from baseline to final follow-up: CMA from 22° to 2°, C2 to T1 distance from 26 mm to 2 mm, clavicle angle from 2° to 0°, CSVL from 4 mm to 0 mm, cervical range of motion from 64° to 79°, and C2–7 SVA from 46 to 13 mm (Figure 4).

## 4. Discussion

CMT occurs in 0.3% to 2% [1], and various causes have been reported, including position in utero [18], birth trauma [19], and compartment syndrome [20]. Histopathologically, adipogenesis, fibrogenesis, and myogenesis were observed in the affected SCM of CMT patients [21]. The mass forms within the SCM around 1 to 3 weeks of age, and parents often discover it as a bump or notice that the affected child’s head is tilted. The head is tilted toward the affected side and also rotated toward the healthy side. Although the majority of patients with CMT heal spontaneously or improve with conservative treatment, there is a possibility of developing residual CMT if the SCM fibrosis is severe. Approximately 10–20% of cases are resistant to conservative treatment [4,7], and those cases should be treated by surgery before school age [10]. However, there are some cases that are undiagnosed or left behind until adolescence or adulthood, resulting in neglected CMT. It is important to note that patients with neglected CMT may present not only with cosmetic problems due to head tilt and SCM protrusion but also with a variety of complaints, such as limited cervical range of motion and neck or shoulder pain [14]. In the present case series, 9/21 patients (42.9%) with neglected CMT complained of numbness around the neck, shoulder, and upper extremities, and those symptoms markedly improved after surgery.

Non-surgical treatment can be applied to patients with CMT, including lifestyle guidance, rehabilitation, or orthosis. However, adolescent or adult patients with neglected CMT are generally resistant to any non-surgical treatments. Although botulinum injections have been tried for CMT [22], there have been no cases of success, with only temporary pain relief in our case series. Surgical treatment should be considered if neglected CMT is residual and symptomatic. Although surgical treatment after the age of 5 years may result in head and face deformities and SCM scarring [10], a systematic review of 220 cases reported that surgical treatment improved appearance, pain, and function in 81% of patients with residual CMT [12]. It was also reported that there was no significant difference in outcomes between patients aged 15 years and older and those aged 15 years and younger [12]. To date, various surgical procedures have been reported in adult patients [13,14,15,16]. Previous studies recommended bipolar release of SCM for patients over 6 years old with neglected CMT or recurrent cases [2,17]. The results of the present study showed that the outcome of unipolar release alone was comparable to that of bipolar release of SCM, even in adolescent or adult patients with neglected CMT. We believe that it was important to dissect the lower ends of the SCM until sufficient mobility of the cervical spine was obtained intraoperatively. In severe or recurrent CMT cases, dissection of tensed platysma muscle or pretrachealis fasciae cervicalis was also required. Additionally, repetitive cervical range of motion training in the early postoperative period is quite important. A previous study recommended a long-term postoperative orthosis [23]. However, prolonged immobilization may lead to reattachment of the SCM and recurrence.

In this study, CMA (head tilt) and C2 to T2 difference (lateral shift of the head) markedly improved after the release of SCM. We have also found that there was an improvement not only in local cervical spine alignment but also in global coronal balance, such as clavicle angle and CSVL. Interestingly, there were also marked improvements in the extension of the cervical spine and C2–7 SVA. To date, there have been no reports evaluating a cervical range of motion between flexion and extension in lateral radiographs or sagittal alignment in neglected CMT. Previous studies have evaluated the outcomes based on head tilt in frontal view or anterior–posterior radiographs and range of motion in rotation and side bending [2,7,10,12,13,14,15,16,17,23]. Nowadays, sagittal alignment of the spine has been highlighted as an important factor affecting patient-reported outcome measures [24,25]. It was reported that C2–7 SVA was significantly correlated with SF-36 physical component scores and the neck disability index [25]. In fact, most of the patients noticed reduced neck and scapula pain, improved numbness in the upper extremities, and better physical balance during lying, sitting, standing, and walking after surgery in the present study. One of the reasons was considered to be their improvements in local and global spinal balance, both in coronal and sagittal alignments, as well as cervical range of motion.

Potential complications of the release of SCM include the possibility of vascular, nerve, and organ injuries, including accessory nerve injury, large vessel injury, thyroid gland injury, tracheal injury, and recurrent nerve injury. In this study, we experienced a case of temporary paralysis of the greater auricular nerve due to retraction, even when no obvious intraoperative injury was observed. The great auricular nerve is a superficial cutaneous branch of the cervical plexus. Injury to the great auricular nerve may result in sensory denervation of the skin of the parotid and periauricular regions [26]. Because the branches of the great auricular nerve are dense at the upper end of the SCM, intraoperative injury is likely to occur, especially when resecting the upper end of the SCM. Therefore, unipolar release of SCM is considered to be a safer technique for reducing the risk of great auricular nerve injury. Unipolar release of SCM may also contribute to wound reduction, shorter operative time, and less bleeding. We emphasize that unipolar release of SCM could achieve favorable outcomes even in adolescent/adult patients with neglected CMT, although bipolar release of SCM had been recommended for neglected CMT in the previous literature [2,17].

There were some limitations to the present study. This study was conducted in a retrospective fashion with a relatively small number of subjects in a single institution since neglected CMT is an extremely rare disease condition, especially in adolescent/adult patients. The number of patients who underwent bipolar release of CMT was only three in this study, and this might affect the statistical results, especially in the group with the bipolar release of CMT. Although bipolar release of CMT is a recommended procedure for neglected CMT in the previous literature, we demonstrated the non-inferiority and benefits of unipolar release of SCM, even in adolescent/adult patients with neglected CMT. In order to verify and compare the clinical outcomes of various surgical procedures and postoperative management for this rare disease condition scientifically, a prospective and multi-center study should be conducted with a large number of adolescent/adult patients with neglected CMT in the future.

## 5. Conclusions

Here, we assessed surgical outcomes between unipolar and bipolar release in adolescent/adult patients with neglected CMT. There were no significant differences in radiographic or clinical outcomes between the two groups. Cosmetic appearance, local and global spinal balance, both in sagittal and coronal alignments, and cervical range of motion improved after surgery. The unipolar release of SCM was considered to be a less invasive procedure that may contribute to wound reduction and reduce the risk of major auricular nerve injury, even in adolescent/adult patients with neglected CMT.

## Figures and Tables

**Table 1:** Demographic characteristics between bipolar release and unipolar release of the sternocleidomastoid.

Demographics	Group of Bipolar Release of Sternocleidomastoid (*n* = 3)	Group of Unipolar Release of Sternocleidomastoid (*n* = 18)	*p*-Value
	Mean ± SD	Mean ± SD	
Age at surgery (years old)	40.0 ± 17.0	32.3 ± 13.1	0.47
Sex	2/3 females	12/18 females	1.00
Height (cm)	162.9 ± 6.1	165.8 ± 6.7	0.69
Weight (kg)	55.0 ± 8.0	59.1 ± 9.1	0.62
Body mass index (kg/m^2^)	20.6 ± 1.8	21.6 ± 3.5	0.84
Affected side (right side)	3/3 cases	12/18 cases	0.53
History of previous surgery for congenital muscular torticollis	1/3 cases	4/18 cases	1.00
Follow-up period (months)	16.0 ± 5.7	10.4 ± 7.6	0.22

**Table 2:** Radiographic parameters between bipolar release and unipolar release of the sternocleidomastoid.

Radiographic Parameters	Group of Bipolar Release of Sternocleidomastoid (*n* = 3)		Group of Unipolar Release of Sternocleidomastoid (*n* = 18)		*p*-Value			
	Preoperative Mean ± SD	Postoperative Mean ± SD	Preoperative Mean ± SD	Postoperative Mean ± SD	P1	P2	P3	P4
Cervicomandibular angle (°)	13.7 ± 2.1	3.3 ± 0.5	15.4 ± 6.5	4.1 ± 2.6	0.10	<0.01	1.00	0.92
C2 to T1 difference (mm)	12.0 ± 4.5	3.3 ± 1.2	14.2 ± 7.7	5.2 ± 4.8	0.10	<0.01	0.69	0.84
Clavicle angle (°)	2.0 ± 1.6	1.3 ± 0.5	2.7 ± 1.3	1.2 ± 0.5	070	<0.01	0.62	0.77
Central sacral vertical line (mm)	7.0 ± 5.4	5.7 ± 2.6	13.5 ± 10.3	5.3 ± 2.8	0.70	<0.05	0.36	0.84
Cervical lordosis (flexion) (°)	−14.3 ± 4.9	−23.7 ± 10.0	−28.6 ± 9.7	−27.4 ± 6.1	0.40	0.61	0.02	0.62
Cervical lordosis (neutral) (°)	7.3 ± 11.0	19.7 ± 9.4	3.6 ± 9.6	7.1 ± 9.4	0.40	0.27	0.77	0.09
Cervical lordosis (extension) (°)	27.7 ± 11.7	42.7 ± 5.2	33.6 ± 11.7	44.1 ± 12.3	0.20	<0.05	0.69	0.77
Cervical range of motion (°)	42.0 ± 7.1	66.3 ± 11.1	62.2 ± 12.2	71.5 ± 13.1	0.10	<0.05	0.02	0.55
C7 sagittal vertical axis (mm)	−15.0 ± 28.2	−4.3 ± 34.4	−12.1 ± 23.3	−13.6 ± 24.8	1.00	0.73	1.00	0.55
C2–7 sagittal vertical axis (mm)	28.7 ± 11.5	14.7 ± 6.3	31.6 ± 11.4	18.4 ± 8.1	0.40	<0.01	0.69	0.62

SD: standard deviation. P1: For the difference between preoperative and postoperative parameters in the group of the bipolar release of the sternocleidomastoid. P2: For the difference between preoperative and postoperative parameters in the group of the unipolar release of the sternocleidomastoid. P3: For the difference in preoperative parameters between groups of the bipolar and unipolar release of the sternocleidomastoid. P4: For the difference in postoperative parameters between groups of the bipolar and unipolar sternocleidomastoid.

## Data Availability

Data sharing is not applicable to this article.
